# Quantitative Analysis of Mother Wavelet Function Selection for Wearable Sensors-Based Human Activity Recognition

**DOI:** 10.3390/s24072119

**Published:** 2024-03-26

**Authors:** Heba Nematallah, Sreeraman Rajan

**Affiliations:** Department of Systems and Computer Engineering, Carleton University, Ottawa, ON K1S 5B6, Canada

**Keywords:** digital signal processing (DSP), human activity recognition (HAR), wavelet transform (WT), wavelet packet transform (WPT), mother wavelet (MW) analysis

## Abstract

Recent advancements in the Internet of Things (IoT) wearable devices such as wearable inertial sensors have increased the demand for precise human activity recognition (HAR) with minimal computational resources. The wavelet transform, which offers excellent time-frequency localization characteristics, is well suited for HAR recognition systems. Selecting a mother wavelet function in wavelet analysis is critical, as optimal selection improves the recognition performance. The activity time signals data have different periodic patterns that can discriminate activities from each other. Therefore, selecting a mother wavelet function that closely resembles the shape of the recognized activity’s sensor (inertial) signals significantly impacts recognition performance. This study uses an optimal mother wavelet selection method that combines wavelet packet transform with the energy-to-Shannon-entropy ratio and two classification algorithms: decision tree (DT) and support vector machines (SVM). We examined six different mother wavelet families with different numbers of vanishing points. Our experiments were performed on eight publicly available ADL datasets: MHEALTH, WISDM Activity Prediction, HARTH, HARsense, DaLiAc, PAMAP2, REALDISP, and HAR70+. The analysis demonstrated in this paper can be used as a guideline for optimal mother wavelet selection for human activity recognition.

## 1. Introduction

The global wearable technology market’s rapid growth has helped people manage their activities to maintain good health. The wearable sensor market is expected to grow at a compound annual growth rate of 13.8% from 2021 to 2028, starting at a value of USD 40.65 billion in 2020 [1]. Wearable technology incorporates electronic devices integrated with sensors worn on the body to obtain real-time data to monitor and track heart rate, sleep hours, and activities. Wearable devices include wristbands, smart watches, smart glasses, fitness trackers, wearable ECG monitors, and biosensors. Some of these devices may have minimal computational resources. Therefore, the demand for precise activity recognition and tracking systems with minimal computational resources has increased.

Deep learning-based human activity recognition (HAR) systems are becoming ubiquitous, but they require a lot of computational resources. On the other hand, conventional approaches have a considerably lower computational burden but require designers with good experience in developing engineered features. Most conventional techniques focus on the signal’s statistical features extracted from time-domain signal data and fast Fourier transform (FFT)-based frequency-domain features. Although such features are helpful, they may need to be more optimal.

Another way to analyze the time signal is to analyze it in the joint time-frequency domain. The wavelet transform (WT) is a powerful method that provides excellent time and frequency localization, making it well suited for multiresolution analysis. The choice of mother wavelet (MW) impacts the information content in the time-frequency domain and, therefore, the features used for classification. Continuous and discrete wavelets are used for multiresolution analysis, where the signal is decomposed into low and high-frequency components for analysis purposes. Another signal processing technique that decomposes a given signal into sub-bands or components using wavelet functions is called Wavelet packet transform (WPT). Unlike discrete wavelet transform (DWT), which only decomposes a signal into low-pass and high-pass components, WPT allows for further decomposition of the high-pass components into narrower frequency bands. This results in a more detailed representation of the signal.

The different types of mother wavelets have different shapes and, therefore, different time and spectral characteristics. Consequently, choosing an MW function similar in shape to the signal of the human activity under consideration is critical. The selection of the MW family significantly affects the HAR accuracy. However, discovering or designing such an optimal mother wavelet for HAR applications remains an open research question.

Various methods exist in signal processing to select the best MW. One such method involves using a fitness function that measures the performance of each candidate wavelet and selecting the one with the highest fitness value as the optimal choice [2]. Fitness functions are used in optimization problems to quantify the quality of a solution. The leading fitness functions used for classification problems are balanced accuracy and F1-score. In comparison, fitness functions for signal recognition problems mainly focus on the shape and characteristics of the signal itself.

The energy-to-Shannon entropy ratio calculation is a valuable measure for achieving the optimal MW selection [3]. This criterion involves selecting the MW with the highest energy-to-Shannon entropy ratio. This ratio takes into account both the energy content and the information content of a signal. By minimizing the Shannon entropy while maximizing the energy, we can select a wavelet that efficiently represents the signal with a minimum number of coefficients.

This research paper presents the quantitative analysis results to select the optimal MW function that accurately captures the desired motion characteristics of the accelerometer signal of each human activity for improved classification performance. Our methodology combined wavelet packet transform (WPT) with the energy-to-Shannon entropy ratio measure and expanded to use it in the classification system. We tested it using two popular classifiers in HAR systems: Decision trees (DT) and support vector machines (SVM). We evaluated six MW families: Haar, Daubechies, Symlets, Coiflets, Biorthogonal, and Reverse Biorthogonal. Our experiments were conducted on eight publicly available ADL datasets: MHEALTH, WISDM Activity Prediction, HARTH, HARsense, DaLiAc, PAMAP2, REALDISP, and HAR70+. The analysis presented in this paper serves as a guide for selecting the optimal MW for HAR systems, highlighting the impact of MW on the classification results. The paper describes a framework for optimal MW selection for HAR using a statistical energy-to-Shannon-entropy ratio. The work used several datasets and multiple classifiers to verify the accuracy of the selected MW. Additionally, the paper delves into an in-depth analysis of various factors present in the datasets, including age group, sensor placement, sensor quality, and sensor units. Experimental verification was conducted utilizing both the energy-to-Shannon-entropy ratio and classification accuracy to confirm the impact of these factors.

The remainder of this paper is organized as follows. In the next Section 2, related works and state-of-the-art are discussed. The proposed methodology and the conducted experiments are described in Section 3. A brief description of the used datasets is presented in Section 4. The results are presented in Section 5, and the main finding and outcome analysis are given in Section 6. Finally, the conclusion and future research directions are provided in Section 7.

## 2. Literature Review

Some early studies compared wavelet transform-based features to time-domain and frequency-domain features [4]. Most of the researchers did not justify their choice of the mother functions. Some of the researchers used the Daubechies mother function. In Ref. [5], using a private dataset, the authors performed a systematic performance analysis of motion-sensor behavior for human activity recognition via smartphones. A combination of time, frequency, and wavelet-domain features was considered. A three-order Daubechies wavelet with five decomposition levels was used to decompose the acceleration data in the vertical direction. Detailed coefficients at the fourth level were used to calculate the wavelet energy. These levels represented a 0.625–2.5 Hz signal band. Daubechies-based dyadic WT and Hidden Markov Model (HMM) were utilized in Ref. [6] to distinguish a high dynamics activity from a low dynamics activity in the accelerometer sensor signals. In Ref. [7], Daubechies 3 Wavelet-autoregression feature extraction was considered for activity recognition from tri-axial acceleration signals. Support Vector Machine (SVM) was chosen as the classifier, and a private dataset with four activities, namely jumping, still, running, and walking, was trained and tested for classification performance. An accuracy of 95.45% compared to 89.42% using traditional time and frequency-domain-based features was obtained. In Ref. [8], a one-level discrete wavelet transforms DWT radial basis neural network (DWT-RBNN) recognition system for elderly daily activity monitoring applications was used to distinguish between walking, jogging, and running activities using Daubechies 10 wavelet as MW for the quadrature mirror filter used in the DWT decomposition process.

Morlet mother function was also considered for HAR. A complex Morlet-wavelet transformed Quaternion (WTQ)-based modified HMMs was proposed in Ref. [9] to improve the accuracy for classifying multi-sensor-based daily locomotion. WPT was proposed in Ref. [10]. A genetic algorithm was used to reduce the number of features, and then feature ranking based on Shapley additive explanations values (SHAP) was utilized to obtain the best features for classification. In the KU-HAR dataset, signal values mainly lie in lower-frequency regions. Various wavelets such as Daubechies, Haar, Symlets, Coiflets, and Bior were considered, features were extracted using each wavelet, and several classifiers’ performances were compared using the features. The Haar mother function achieved the best accuracy.

Instead of using a MW, the concept of multiresolution using the high-pass and low-pass filters and decimation has also been considered in the literature. In Ref. [11], two novel approaches to feature extraction based on vector autoregression and undecimated discrete wavelet transform (DWT), together with four different classifiers, were studied. In Ref. [12], wavelet energy spectrum features of acceleration for activity recognition with an ensemble-based filter feature selection (EFFS) approach was utilized, and the concept was tested on a private dataset. The feature set with wavelet energy spectrum features improved the discrimination of activities. In Ref. [13], 34 different motor activities in a customized training program for people with Parkinson’s disease were obtained using wearable IMU sensors. Time, frequency domains, discrete wavelets, and several classifiers were studied. Although no mention of the type of the MW was made in the paper, the wavelet-based extracted features, such as the sum of wavelet coefficients, the sum of squared wavelet coefficients, and the energy value of these coefficients, were listed. The model in Ref. [14] extracted data via a hierarchical feature-based technique. These features included time, wavelet, and time-frequency domains. Stochastic gradient descent (SGD) was then introduced to optimize the features. The selected parts with optimized patterns were further processed by multi-layered kernel sliding perceptron to develop adaptive learning to classify human activities. The proposed model was experimentally evaluated on the PAMAP2 dataset, and an accuracy rate of 94.16% was reported. The paper has no information about the used mother function or the features extracted from the wavelet decomposition.

Some research papers explored the importance of using wavelet analysis with HAR to distinguish between two/three activities. In Ref. [15], wavelet energy of the vertical acceleration data was utilized to improve the recognition accuracy between climbing upstairs and downstairs activities. In Ref. [16], continuous wavelet transformation (CWT) of the accelerometer data was employed to detect sit-to-stand movement. A custom wavelet was generated from a representative sample sit-to-stand movement over segments of 0.5–5 s, and the acceleration signal’s vector magnitude was calculated. The paper did not explain the choice of representative sample or the segments’ lengths. In Ref. [17], a WPT-based feature extraction technique was applied to accelerometer and gyroscope data to differentiate between high and low-resistance biking, running, and walking. Statistical features, along with WPT features, such as mean, variance, skewness, kurtosis, and entropy, were also used.

In Ref. [18], CWT was utilized to encode the time series of sensor data as multi-channel images. It was used as an input to a spatial attention-aided convolutional neural network (CNN). Mutual Information (MI), Relief-F, and minimum redundancy maximum relevance (mRMR) filter-based methods were used for feature selection. Experiments on five publicly available HAR datasets showed an increase in the overall prediction accuracy of 0.18–1.04% compared to state-of-the-art approaches. The authors of Ref. [19] presented a hierarchical classification framework based on Haar-based wavelets and adaptive pooling for activity recognition and fall detection. The sisFall, a publicly available dataset, was used to determine the best observation window size of 3 s. The results showed a weighted F1 score of 94.67%.

Multiresolution time-frequency analysis of received signal strength (RSS) between the wearable sensors was considered in Ref. [20]. The embedded inertial-sensor signal data are decomposed using Haar-based DWT and the empirical mode decomposition (EMD) techniques to reach a maximum classification accuracy of 99.63%.

**Figure 1:**
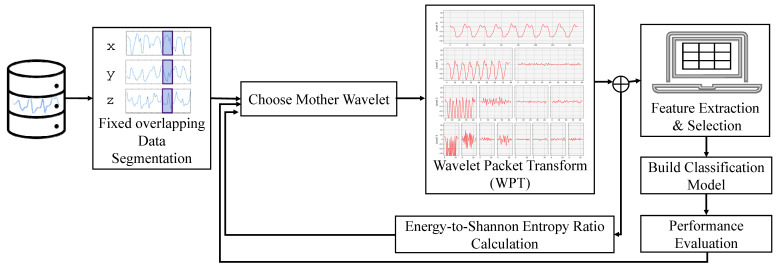
The system’s full pipeline.

There was minimal research on defining a fitness function for optimal mother wavelet selection. The research in Ref. [2] used a technique formulated using parametric analysis of variance (ANOVA) and genetic algorithm (GA). The work in Ref. [21] aimed to find the optimal MW and wavelet decomposition level when denoising the Doppler cardiogram (DCG). The paper presented quantitative analysis results by evaluating the signal-to-noise-ratio (SNR) efficiency of the denoised signals obtained using the wavelet thresholding method. The paper reported db9 and sym9 as DCG’s most efficient mother wavelet functions. On the other hand, the work in Ref. [22] found that db4 is the most suitable MW for EEG frequency band decomposition using linear discriminant analysis (LDA) for feature selection. The pipeline of the proposed system is shown in Figure 1.

## 3. Methodology

This section will cover the optimal MW selection methodology for human activity recognition. In Section 3.1, wavelet packet transformation WPT of the accelerometer time series data for human activities is introduced. In Section 3.2, discrete mother wavelets and their respective characteristics are discussed. The energy-to-Shannon rate ratio measure, which is introduced as a fitness function for optimal selection, is introduced in Section 3.3. Feature extraction and classification algorithms are described in Section 3.4.

### 3.1. Wavelet Packet Transform (WPT)

The wavelet packet transform (WPT) is an extension of the discrete wavelet transform (DWT) technique. The WPT overcomes the limitations of fixed time-frequency decomposition in DWT. This method provides a complete level-by-level signal decomposition by decomposing a time signal into low-frequency approximation and high-frequency detail signals. One of the critical advantages of WPT is its ability to combine different decomposition levels to achieve an optimal time-frequency representation of the original signal. A wavelet packet $\psi^{i}\left(t\right)$ comprises a set of linearly combined conventional wavelet functions and inherits orthonormality and time-frequency localization properties from their corresponding wavelet functions ψ(t) as in (1). A wavelet packet $\psi_{j,k}^{i}\left(t\right)$ can be represented as

(1) $$\psi_{j,k}^{i}\left(t\right)=2^{-j/2}\cdot \psi^{i}\left(2^{-j}t-k\right)$$

where integers i,j and *k* are the modulation, scale, and translation parameters, respectively, and

(2) $$\psi^{2i}\left(t\right)=\frac{1}{\sqrt{2}}\sum_{k=-\infty }^{\infty }h\left(k\right)\psi^{i}\left(t/2-k\right)$$

(3) $$\psi^{2i+1}\left(t\right)=\frac{1}{\sqrt{2}}\sum_{k=-\infty }^{\infty }g\left(k\right)\psi^{i}\left(t/2-k\right)$$

where h(k) and g(k) are the high-frequency and low-frequency discrete filters, respectively, that are associated with the scaling function and the mother wavelet function.

**Figure 2:**
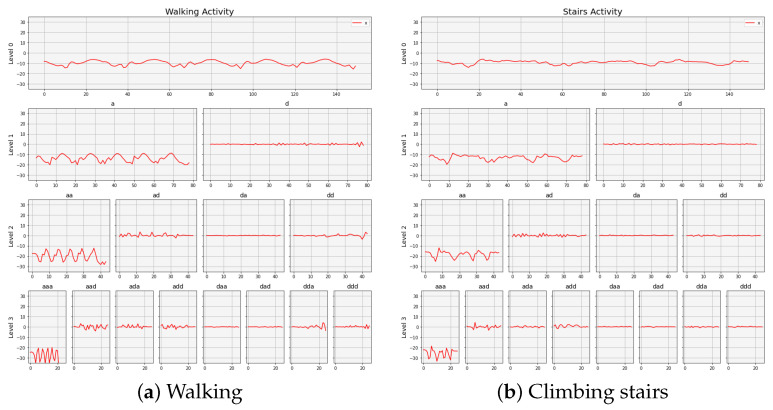
Three-level wavelet packet decomposition of a sample signal data collected from an accelerometer attached to the chest of the subject.

The three-level db9-based wavelet packet decomposition of a sample walking signal data and climbing stairs signal data are shown in Figure 2a,b. It is worth noting that different activities exhibit different frequency distributions in their acceleration signals.

### 3.2. Wavelet Functions

Since the first introduction of wavelets by A. Haar in 1909 [23], several groups working independently developed several wavelet function families [3]. Each wavelet family is represented by a wavelet mother function ψ(t) and a scaling function ϕ(t). The scaling function is needed to capture low and zero-frequency components in the input signal. The most common families are Daubechies, Coiflets, Symlets, and Biorthogonal. Under each family, there are several different wavelet functions [23].

**Figure 3:**
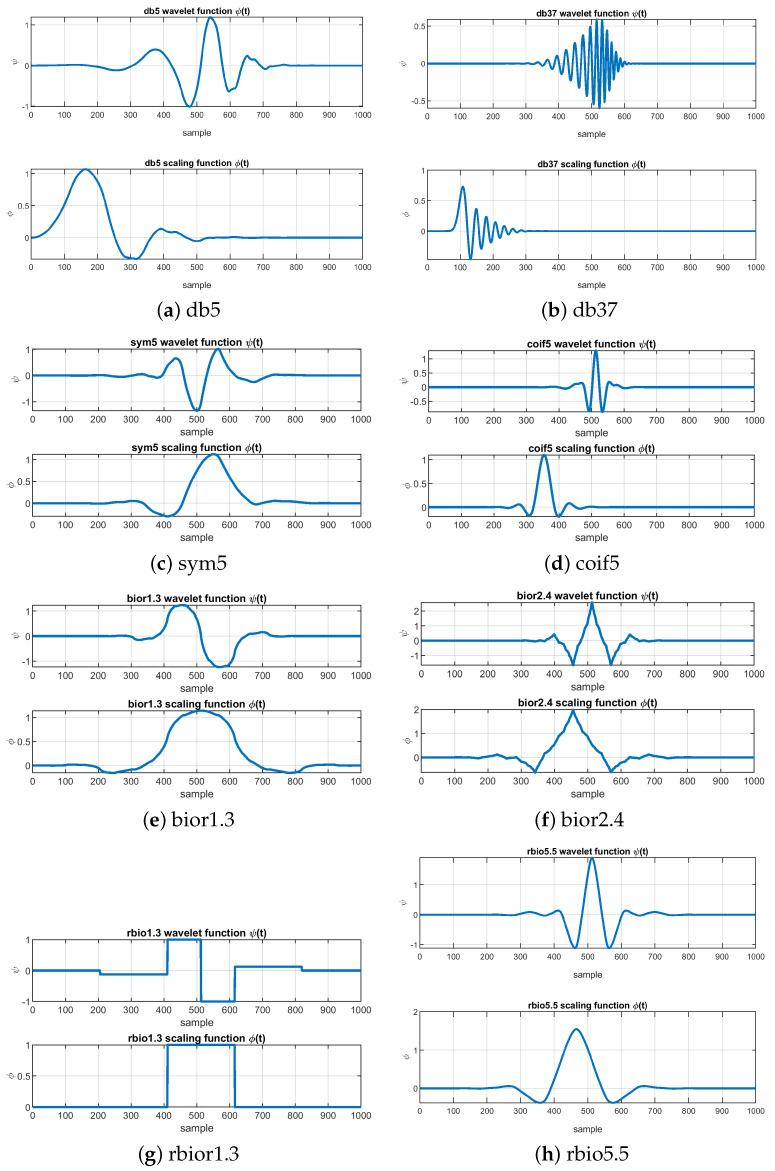
Different mother wavelet functions and scaling functions.

The Haar wavelet is the first-order Daubechies wavelet, which resembles a step function. The order of the Daubechies functions denotes the number of vanishing moments. The larger the order of the filter, the better the frequency localization of the decomposition. Coifman wavelets are orthogonal compactly supported wavelets with the most vanishing moments. The Biorthogonal wavelet family exhibits the linear phase property using two wavelet functions, one for decomposition and the other for reconstruction. The Symlets are near symmetric, orthogonal, and compactly supported wavelets. Some of these wavelet mother functions and scaling functions are shown in Figure 3. More details about MW and the effect of the vanishing moments and the scaling function can be found on Ref. [23].

When selecting an MW, the number of vanishing moments is crucial. This factor determines the smoothness and regularity of the wavelet function and its ability to analyze signals with varying characteristics. In wavelet analysis, vanishing moments indicate the number of moments of a wavelet function that are zero. Wavelet functions with more vanishing moments effectively analyze signals with smooth variations. In contrast, those with fewer vanishing moments are better suited for signals with sharp transitions. The list of selected MW with the number of vanishing moments is shown in Table 1. It is worth noting that the number following the mother wavelet’s name often indicates the number of vanishing moments for the wavelet.

### 3.3. The Energy-to-Shannon Entropy Ratio

When analyzing a signal in the time-frequency domain, it is crucial to determine the best-suited mother wavelet [24]. This work uses the energy-to-Shannon Entropy ratio as a fitness function for optimal mother wavelet selection. A fitness function is any mathematical function that determines the effectiveness of a solution in meeting a specific objective. The optimal or near-optimal solution to the problem is the one with the highest fitness score. This work aims to find a mother wavelet that can extract the most significant amount of energy while minimizing the Shannon entropy.

The energy of a signal *E* is a mathematical measure of the total magnitude of the signal over time. It represents the total power of the signal and is typically calculated by finding the integral of the signal’s squared magnitude over time. As shown in (4), the E(n) is energy at decomposition level *n*.

(4) $$E\left(n\right)=\sum_{i=1}^{m}{\left|C_{n,i}\right|}^{2}$$

where $C_{n,i}$ is the *i* th wavelet coefficient at level *n* and *m* is the number of wavelet coefficients.

In contrast, the Shannon entropy of a signal is calculated based on the probability distribution of the signal’s frequencies. A signal with high entropy contains a smaller amount of frequency probability distribution. The Shannon entropy at decomposition level *n* is given by (5).

(5) $$S_{\mathrm{entropy}\;}\left(n\right)=-\sum_{i=1}^{m}P_{i}\times \log P_{i}$$

where $P_{i}$ is the distribution of the energy probability for the wavelet coefficients as shown in (6)

(6) $$P_{i}=\frac{{\left|C_{n,i}\right|}^{2}}{E(n)}$$

where $\sum_{i=1}^{m}P_{i}=1$. The value of $P_{i}\times \log P_{i}$ is assumed zero when $P_{i}=0$.

Then, the energy-to-Shannon entropy ratio criterion is used, which means maximizing the amount of energy while minimizing the Shannon entropy of the corresponding wavelet coefficients.

(7) $$\xi \left(n\right)=\frac{E(n)}{S_{\mathrm{entropy}\;}\left(n\right)}$$

### 3.4. Signal Classification

The process of selecting the best wavelet for signal classification involves utilizing cross-validation. To apply cross-validation for mother wavelet selection, after defining the set of candidate mother wavelets mentioned earlier, we split the available dataset into k folds; in our case, we choose k equal to ten. Then, for each candidate mother wavelet, a classifier is trained using the extracted set of features. Then, we evaluate the classifier’s performance on the test fold using a suitable performance metric, such as accuracy, precision, or recall. The top-performing mother wavelet is considered the optimal choice.

#### 3.4.1. Feature Extraction

We computed statistical features for each WPT-transformed signal data sub-band to expand the optimal wavelet selection into the classification performance. Each sub-band’s extracted features are listed in Table 2.

#### 3.4.2. Classification Algorithms

To select the mother wavelet function that results in the optimal analysis of the input activity data signal, decision tree (DT) and SVM algorithms were used as the classifier. The generated features from each sub-band of the wavelet transform are grouped and used as input for the classifier.

A decision tree (DT) is a supervised classification technique with a hierarchical structure. It consists of a root node, branch nodes, and leaf nodes, where each non-leaf node represents a feature or attribute, each branch represents a decision or rule, and each leaf denotes a resulting classification category. In this project, we employed the C4.5 algorithms for tree splitting. The primary benefits of the DT classifier include its interpretability and ability to handle missing, categorical, and quantitative values. However, it is worth noting that DT can be unstable, and it is challenging to manage tree size.

The support vector machine (SVM) works by finding the best hyperplane that separates the data points of different classes. The hyperplane was chosen to maximize the margin between the closest data points of different classes. The data points closest to the hyperplane are referred to as support vectors, where the SVM acquires its name. SVMs are one of the most effective algorithms for classification tasks.

#### 3.4.3. Classification Performance Measures

To provide an analytical comparison of the different mother wavelets, $f1-score\left(c_{j}\right)$ was used as the metric. This metric $f1-score\left(c_{j}\right)$ is the harmonic mean of precision and recall. Precision, $P(c_{j})$ is the percentage of relevant classification instances, while recall $R(c_{j})$ is the percentage of relevant total results correctly classified. Mathematical equations for class-wise precision, recall, and f1−score are given below.

(8) $$P\left(c_{j}\right)=\;\frac{TP\left(c_{j}\right)}{TP\left(c_{j}\right)+FP\left(c_{j}\right)}$$

(9) $$R\left(c_{j}\right)=\;\frac{TP\left(c_{j}\right)}{TP\left(c_{j}\right)+FN\left(c_{j}\right)\;}$$

(10) $$f1-score\left(c_{j}\right)=\frac{2P\left(c_{j}\right)R\left(c_{j}\right)}{P\left(c_{j}\right)+R\left(c_{j}\right)}$$

where $TP\left(c_{j}\right)$ is the number of True Positive instances of class $c_{j}$; $FP\left(c_{j}\right)$ is the number of False Positive of class $c_{j}$ and $FN\left(c_{j}\right)$ is the number of False Negative of class $c_{j}$.

## 4. Datasets

Eight diverse datasets containing a broad range of activities were utilized to accomplish a comprehensive performance evaluation. These datasets also feature human activity data from various age groups and facilitate examining how subject age impacts the selection of the mother wavelet. During the preprocessing of the activity’s time series data, we implemented a fixed segmentation technique. This approach involves dividing the time series sensor data into fixed windows of specified time intervals, which enables the extraction of feature sets from each window. The use of fixed segmentation is particularly beneficial when working with multiple datasets, as it simplifies the comparison and analysis of data. Moreover, automating data processing is made easier with the use of this technique. A fixed segmentation duration of 2.5 s is commonly used [25]. As a result, we utilized a fixed segmentation window of 2.5 s across all the datasets. After applying a downsampling technique with a sampling rate of 50 Hz due to the varying sampling rates between the different datasets, the feature set outlined in Section 2 was extracted from each segment. The datasets utilized for this purpose are as follows:

### 4.1. WISDM

**Figure 4:**
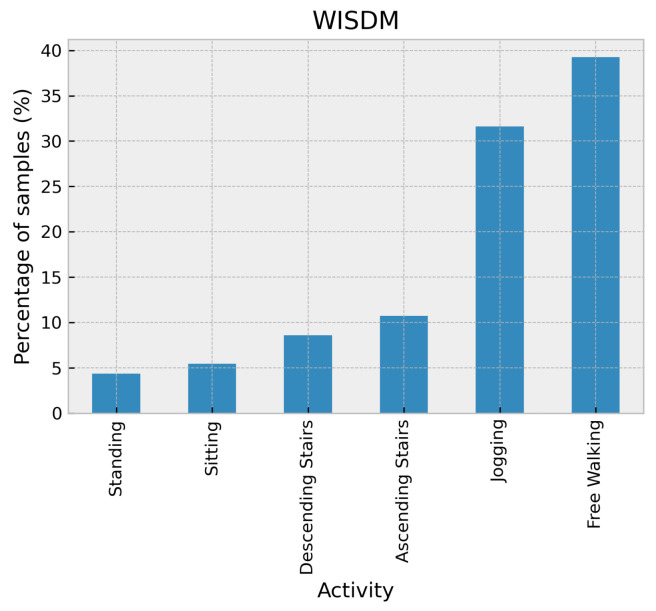
Distribution of activities in the WISDM dataset.

The Wireless Sensor Data Mining (WISDM) dataset [26] was collected using several types of Android Phones, including the Nexus One, HTC Hero, and Motorola Backflip, which were placed in the leg front pant pocket of the subject. It contains tri-axial accelerometer data from 29 subjects who performed activities in a controlled, supervised environment for predefined specific periods with a sampling rate of 20 Hz. The activities in the WISDM dataset include six activities: walking, jogging, ascending stairs, descending stairs, sitting, and standing. As shown in Figure 4, this dataset is imbalanced where only two activities, walking and jogging, are more than half the collected data.

### 4.2. HARSense

**Figure 5:**
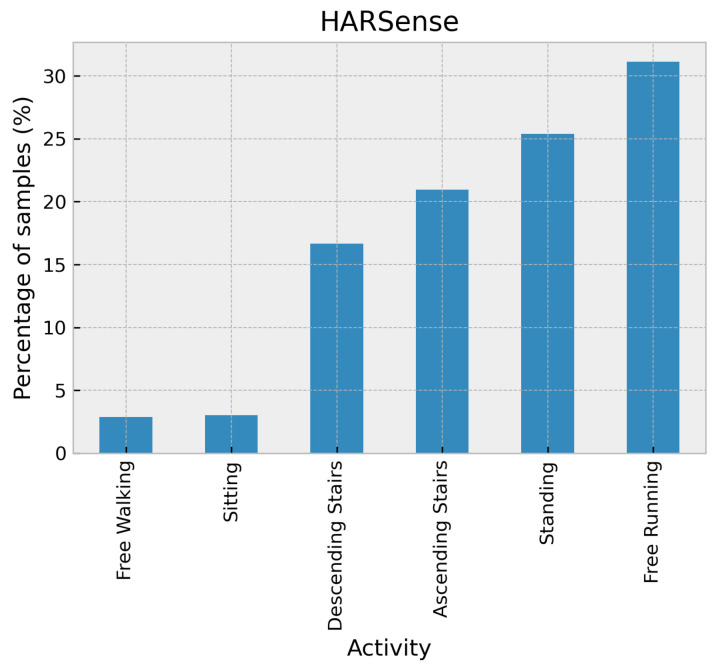
Distribution of activities in the HARSENSE dataset.

The HARSense dataset [27] comprises activity data obtained from a smartphone accelerometer sensor on each subject’s waist and front pockets with a sampling rate of 50 Hz. The smartphones utilized were the Poco X2 and Samsung Galaxy A32s. The activities performed included walking, standing, upstairs, downstairs, running, and sitting and were completed by 12 participants in a closely observed, controlled setting. This dataset is also imbalanced, as shown in Figure 5.

### 4.3. HARTH

**Figure 6:**
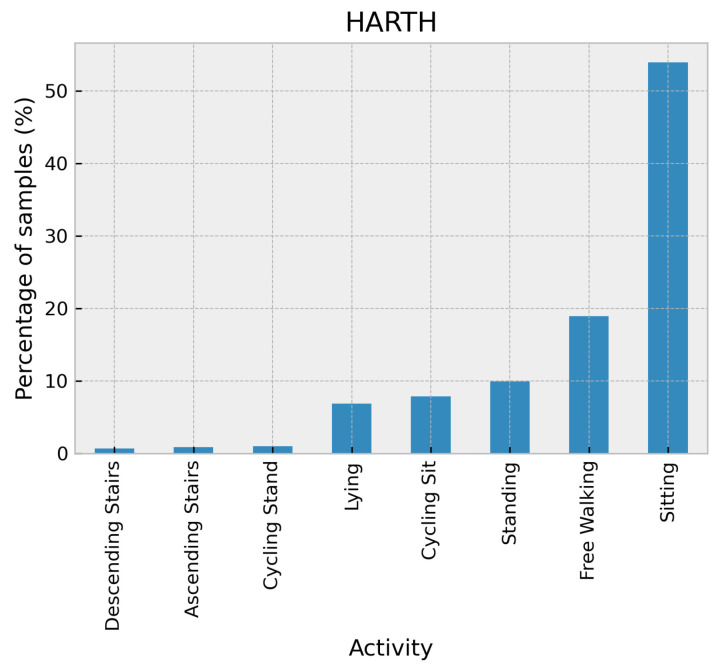
Distribution of activities in the HARTH dataset.

The HARTH dataset [28] contains recordings of 22 participants wearing two 3-axial Axivity AX3 accelerometers for around 2 h in a free-living setting. One sensor was attached to the front of the right thigh and the other to the lower back. The sampling rate was set to 50 Hz. To annotate the activities performed, video recordings from a chest-mounted camera were used to analyze each frame thoroughly. The sampling rate of the accelerometer data (in terms of several g) is 50 Hz. The dataset contains the following activities: walking, running, stairs (ascending), stairs (descending), standing, sitting, lying, cycling (sit), and cycling (stand). The distribution of activities in the HARTH dataset is shown in Figure 6.

### 4.4. HAR70+

**Figure 7:**
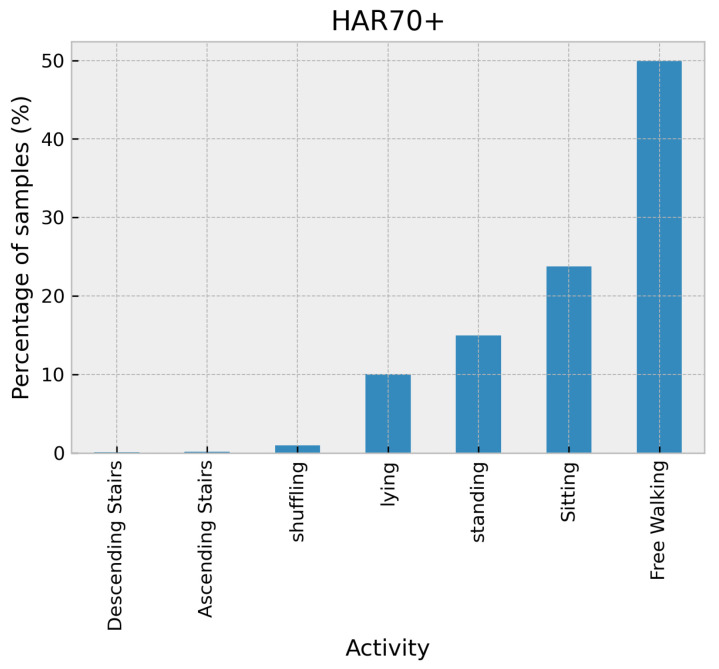
Distribution of activities in the HAR70+ dataset.

The HAR70 dataset [29] gathered data from a group of 18 older adults ranging in age from 70 to 95. Four of these participants used a walker for all walking activities, while one used a walking stick when activities were performed outside. Each person wore two accelerometers and a camera mounted on their chest and completed multiple repetitions of common daily living activities such as walking, standing, sitting, and lying down. The Axivity AX3 accelerometers were placed on the lower back and right thigh and recorded at a sampling frequency of 50 Hz with a range of ±8 g. The dataset comprises mostly of walking activity samples, accounting for over 50% of the total dataset. The remaining samples are for static activities such as sitting, standing, and lying down, taking up nearly half of the dataset. However, the dataset only contains less than 2% of the samples for physically demanding activities such as climbing stairs and shuffling, as illustrated in Figure 7.

### 4.5. MHEALTH

**Figure 8:**
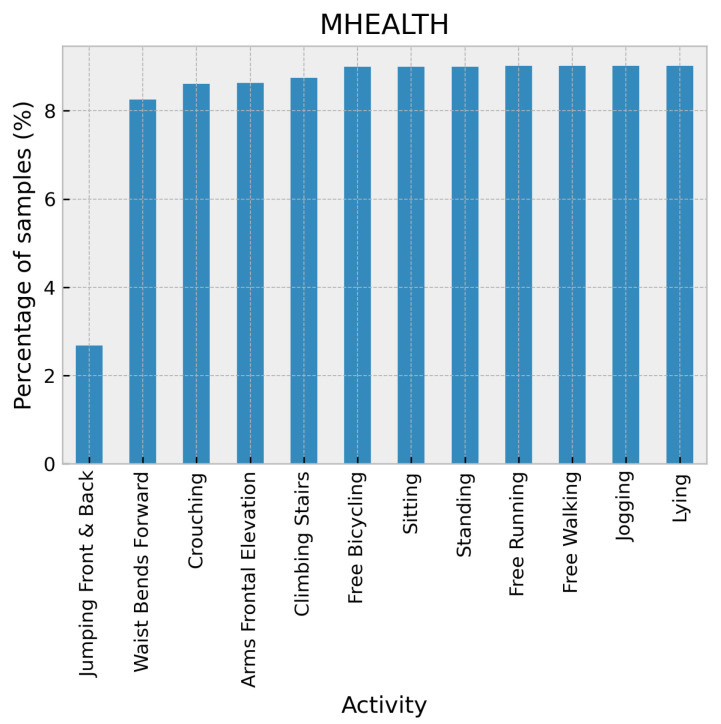
Distribution of activities in the MHEALTH dataset.

The MHEALTH dataset [30] comprises body motion and vital signs recordings for ten volunteers performing 12 physical activities. Shimmer2 wearable sensors were placed on the subject’s chest, right wrist, and left ankle with a sampling rate of 50 Hz. The activities were collected in an out-of-lab environment with no constraints on execution except trying their best when executing them. The activity set consists of standing still, sitting and relaxing, lying down, walking, climbing stairs, waist bends forward, frontal elevation of arms, knees bending (crouching), cycling, jogging, running, and jumping front and back. The distribution of the activities in the MHEALTH dataset is shown in Figure 8.

### 4.6. PAMAP2 Dataset

**Figure 9:**
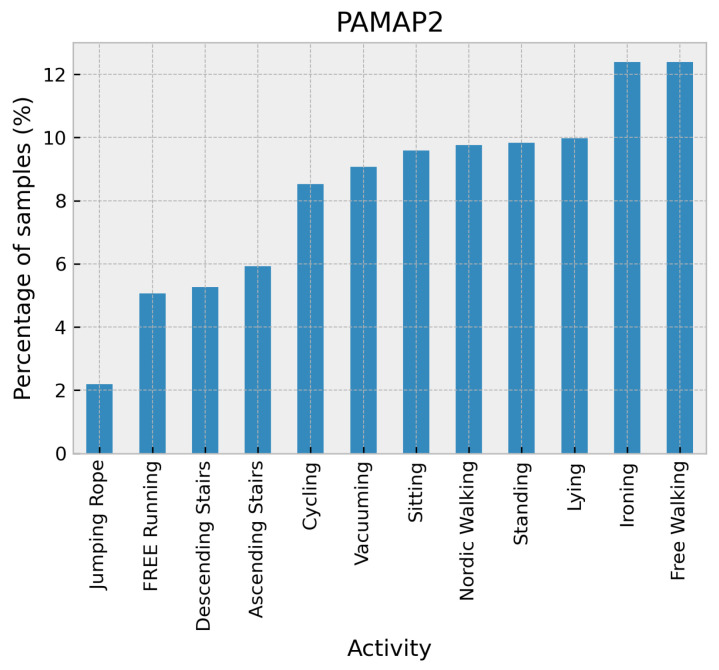
Distribution of activities in the PAMAP2 dataset.

Physical Activity Monitoring (PAMAP2) [31] utilized three Colibri wireless inertial measurement units with a sampling frequency of 100 Hz were used. These sensors were placed on the dominant arm’s wrist, the chest, and the ankle of the dominant side. The data collection involved nine subjects performing 12 different activities, with some of them performing six additional activities. The activities included lying, sitting, standing, walking, running, cycling, Nordic walking, watching TV, computer work, car driving, ascending stairs, descending stairs, vacuum cleaning, ironing, folding laundry, house cleaning, playing soccer, and rope jumping. A distribution of these activities in the PAMAP2 dataset can be seen in Figure 9.

### 4.7. REALDISP

**Figure 10:**
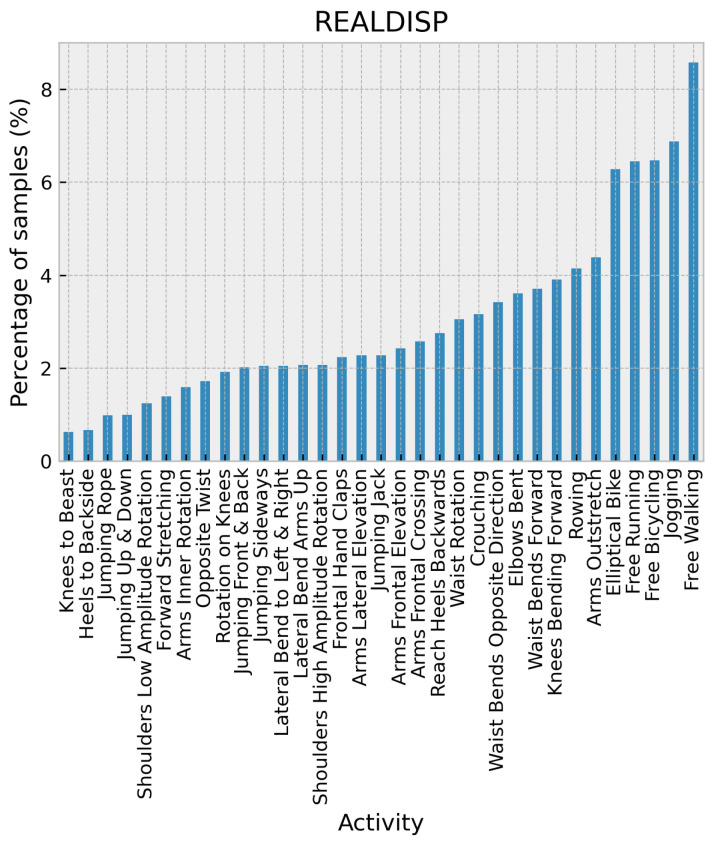
Distribution of activities in the REALDISP dataset.

The realistic sensor displacement dataset [32] was gathered by placing nine Xsens inertial measurement units throughout the subject’s body. These units were placed on the left calf, right calf, left thigh, right thigh, left lower arm, right lower arm, left upper arm, right upper arm, and back. The data were sampled at 50 Hz. Various warm-up, fitness, and cool-down exercises are part of the dataset. The dataset also includes whole-body movements such as walking, jogging, running, jumping up, jumping front and back, jumping sideways, jumping legs/arms open/closed, jumping rope, rowing, elliptic bike, and cycling. Additionally, it contains body part-specific activities that focus on the trunk, such as trunk twist (arms outstretched), waist rotation, waist bends (reaching foot with opposite hand), reaching heels backward, lateral bend, Lateral bend arm up, repetitive forward stretching, upper trunk and lower body opposite twist, trunk twist (elbows bent), and waist bend forward. Upper extremity exercises are also included, such as shoulders high amplitude rotation, shoulders low amplitude rotation, Arms inner rotation, arms lateral elevation, rotation on the knees, arms frontal elevation, frontal hand claps, and arms frontal crossing. Finally, lower extremity exercises such as knees alternatively raised to the chest, heels alternatively raised to the backside, knees bending (crouching), and knees alternatively bending forward are also part of the dataset. The activity distribution is illustrated in Figure 10.

### 4.8. DaLiAc

**Figure 11:**
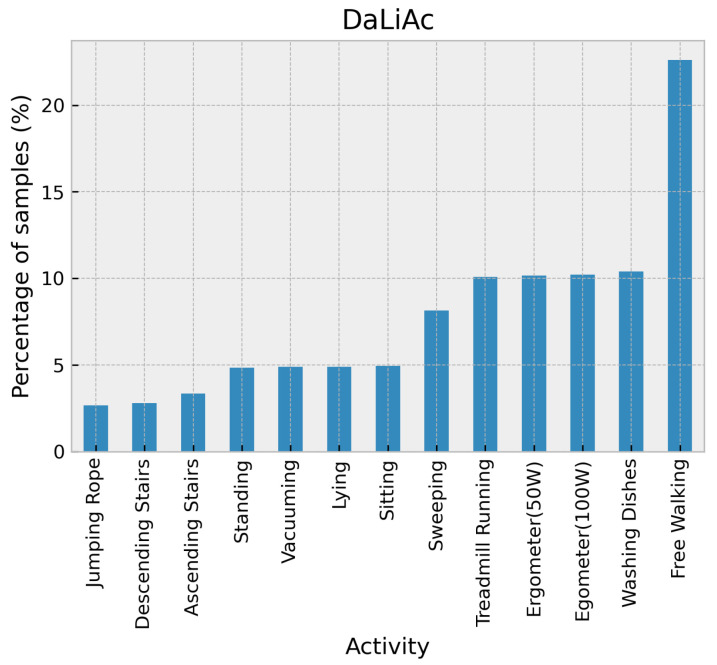
Distribution of activities in the DaLiAc dataset.

Daily Life Activities (DaLiAc) dataset [33] contains data on daily life activities collected with inertial sensors. Thirteen activities performed by 19 participants were recorded using four sensor nodes placed on the right hip, chest, right wrist, and left ankle at a sampling rate of 204.8 Hz. The activities include sitting, lying, standing, washing dishes, vacuuming, sweeping, walking, ascending stairs, descending stairs, treadmill running, bicycling on the ergometer (50 W), bicycling on the ergometer (100 W), and rope jumping. Activity distribution in the DaLiAc dataset is shown in Figure 11.

## 5. Results

In this work, the proposed HAR platform was developed using Python 3.7 with scikit-learn and NumPy libraries. The system was trained and tested on an Asus laptop i7-intel CPU running at 2.8 GHz with an Nvidia GTX 1050 GPU with 8 GB RAM.

**Figure 12:**
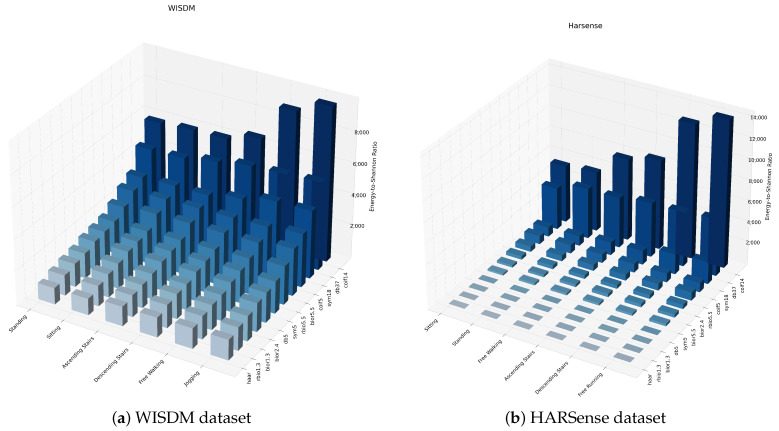
Energy-to-Shannon ratio of each activity data of WISDM and HARSense datasets.

The results of the wavelet decomposition analysis for optimal mother wavelet selection for the WISDM dataset have been investigated as the first stage in this study. As shown in Figure 12a, the coif14 and db37 MWs have been found to generate the highest energy-to-Shannon ratios, while the Haar and rbio1.3 MWs produced the lowest ratios. The ranking of the MWs remained consistent across all activities, with minimal variations between them. Furthermore, upon comparing the selection of mother wavelets for the HARSense dataset, the same optimal choices as those of the WISDM dataset were confirmed despite having a comparatively higher number of worst-performing MWs, as shown in Figure 12b.

**Figure 13:**
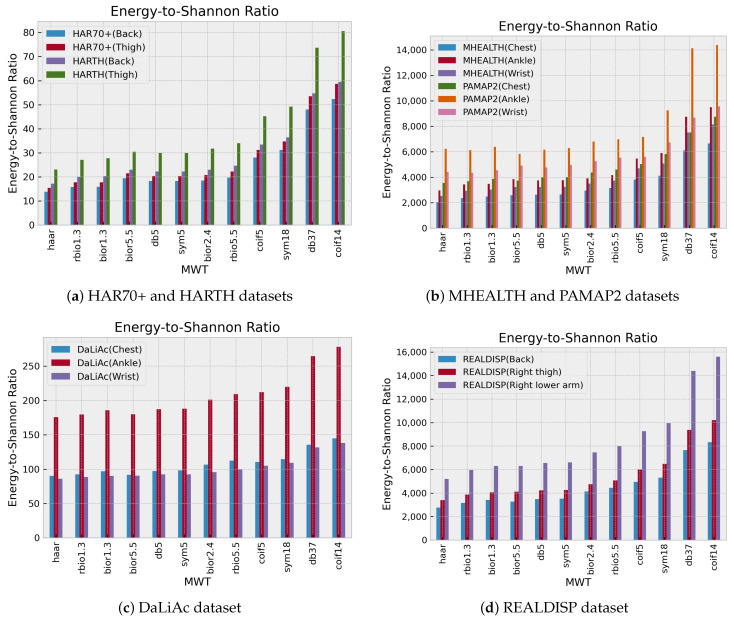
Comparison of the energy-to-Shannon ratio of each mother wavelet.

In addition, the analysis of the average entropy-to-Shannon entropy ratio of each sensor of both HAR72+ and HARTH, DaLiAC, MHEALTH, PAMAP2, and REALDISP datasets has also been conducted as shown in Figure 13. Although these datasets have a more comprehensive range of different activity types that involve physical activity, the analysis revealed that coif14 and db37 are optimal. At the same time, Haar and rbio1.3 are not recommended for human activity recognition.

Additionally, upon examining the ratio range between DaLiAc, MHEALTH, and PAMAP2 datasets, it was discovered that DaLiAc’s ratio range is less than 20% compared to the other two. This can be attributed to the fact that the accelerometer data unit is measured in *g* in DaLiAc, while m/s^2^ is utilized in the other two datasets. Similar reasoning accounts for the narrow ratio range observed in the HAR70+ and HARTH datasets.

**Figure 14:**
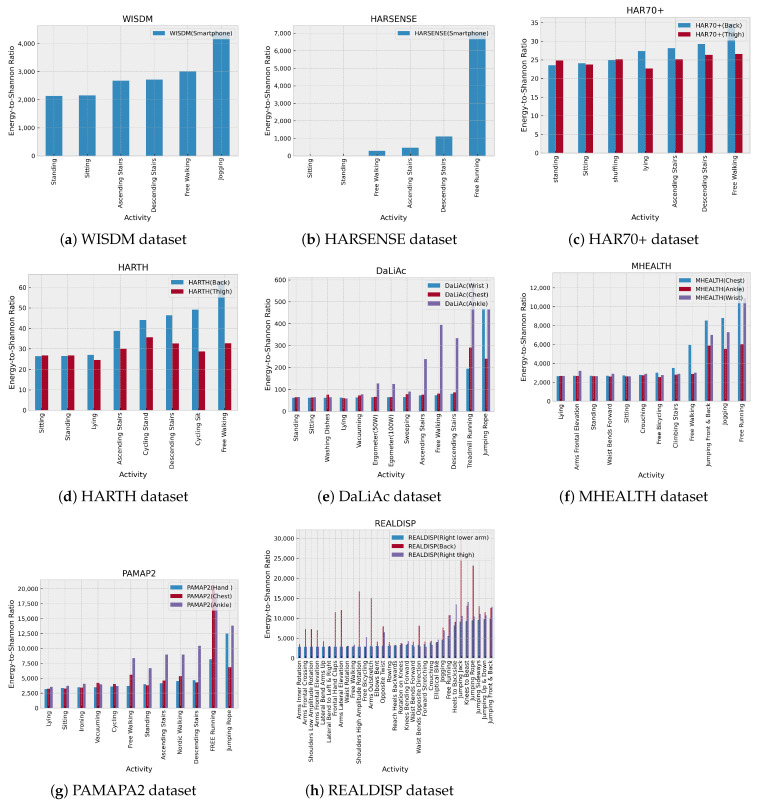
Comparison of the energy-to-Shannon ratio of each mother wavelet for HAR70+, HARTH, and REALDISP datasets using coif14-based wavelet packet transform.

As part of our research, we aim to determine the impact of optimal mother wavelet selection on the classification performance of human activities. Our initial focus has been on evaluating the significance of the feature energy-to-Shannon entropy ratio, using the optimal wavelet coif14 to analyze separability between different activity classes. In Figure 14, we present the averaged ratio of each activity using coif14-based wavelet packet transform. Our findings for the WISDM dataset show that the average range of the energy-to-Shanon entropy ratio increases with higher activity dynamics, with the lowest range observed for static activities such as sitting and standing. Dynamic activities like jogging and walking have a higher ratio range, with an increase of approximately 30% observed as walking dynamics transition to jogging.

The HARSENSE dataset analysis also yielded similar findings. However, climbing stairs activities exhibited a higher average ratio compared to walking activities. This discrepancy could be attributed to variations in the execution of the activity across different datasets, or it could imply the necessity for more significant features to differentiate between comparable dynamics in activities.

It is important to note that the above findings may not necessarily apply to the HAR70+ dataset. While dynamic activities still have a higher average ratio than static activities, it is worth mentioning that lying-down activities have a higher ratio than shuffling activities for both sensors attached to the thigh and the back of the subject. When analyzing the HAR70+ dataset, it is crucial to take into account the unique characteristics of the subjects who volunteered for data collection. This is the only dataset available where older individuals ranging from 70 to 95 years old participate in conducting activities, where some of them use walking aids, which may indicate limited mobility. In contrast, all other datasets were collected from healthy individuals between the ages of 20 and 35. Thus, the HAR70+ dataset is possibly the only dataset that can be used for aging-in-place studies, and if others are used, they may result in providing biased results.

A comprehensive representation of human motion may be obtained when sensors are placed on the ankle or the thighs. When it comes to physical activities such as treadmill running, free running, and various types of jumping, the sensor attached to the subject’s thigh or ankle records a more exhaustive range of time series data compared to the one attached to the chest or back. This is because these activities are predominantly driven by movements of the thigh or ankle, which have broader variations in the frequency range than the chest movements. Datasets such as HARTH, DaLiAc, MHEALTH, PAMAP2, and REALDISP confirm this observation through the analysis presented in this work.

Optimal mother selection has been achieved by evaluating the classification performance of a human activity recognition system based on wavelet decomposition analysis using two different mother wavelets, namely coif14 and Haar, which were selected as the optimal and least suited MWS, respectively, as discussed earlier. The system’s performance has been evaluated in Table 3. These results agree with the earlier finding that coif14 leads to higher balanced accuracy and average F1-score for decision tree and support vector machine classifiers. At the same time, based on this paper’s findings, Haar MW is not recommended for human activity recognition despite its broad usage in the literature.

## 6. Discussion

Significant variations exist in the energy-to-Shannon entropy ratios across different datasets. This could be attributed to two potential factors. First, the accelerometer signal’s unit likely contributes to the range of diverse ratios. When measured in m/s^2^, the signal exhibits large time-domain values of ±g m/s^2^. However, when measured in g, the time-domain signal values fall between −1 and +1. Equation (4) shows that energy corresponds to the total signal magnitude over time. Accordingly, a larger magnitude results in higher energy and, consequently, a higher $S_{\mathrm{entropy}}$ ratio. This correlation is evident in the differences observed in the ratio range between datasets with accelerometer signals measured in g versus m/s^2^.

Second, a low energy-to-Shannon ratio (ξ) can be caused by either a low signal energy (*E*) or high Shannon entropy ($S_{\mathrm{entropy}}$) (see Equation (7)). Conversely, a high ξ can be the result of high *E* or low $S_{\mathrm{entropy}}$. A signal of superior quality can be represented by only a few wavelet coefficients with a narrow distribution (low entropy), resulting in high $S_{\mathrm{entropy}}$. On the other hand, a signal with a high noise level may contain numerous wavelet coefficients with a widespread probability distribution for each coefficient, leading to high entropy and, therefore, a low ratio. As a result, the energy-to-Shannon entropy ratio could potentially serve as an indicator of the signal quality and, in turn, the quality of the inertial sensors used in the data collection process of each dataset.

Furthermore, this research has revealed that the range of activities captured in the dataset does not affect the selection of the ideal mother wavelet, as our work studied various datasets containing diverse activities with varying distributions. Moreover, the energy-to-Shannon entropy ratio can provide insight into the movement patterns and dynamics of the body part’s leading motion during the activity using multiple sensor placements. These observations are valuable in developing human activity recognition systems through wavelet decomposition analysis.

## 7. Conclusions

This paper demonstrated the role played by the optimal mother wavelet selection in classifying different activities in a HAR system. The study used eight publicly available datasets, namely MHEALTH, WISDM Activity Prediction, HARTH, HARsense, DaLiAc, PAMAP2, REALDISP, and HAR70+, where only the accelerometer sensor signal was considered. We examined six different mother wavelet families with varying numbers of vanishing points: Haar, Daubechies, Coiflets, Symlets, Biorthogonal wavelets, and Reverse Biorthogonal. Coiflet was found to be the optimal mother wavelet function to discriminate activities. At the same time, Haar was the least suitable for such activity recognition.

As part of future studies, signal quality analysis will be conducted, and signal processing algorithms for improving signal quality may be incorporated as part of the preprocessing phase. A detailed feature analysis will also be conducted. In addition, uncertainty analysis will be carried out to classify various activities.

## Figures and Tables

**Table 1:** Used Mother wavelet functions.

Wavelet Family	Mother Wavelet with Vanishing Moments
Haar	Haar
Daubechies	db5,db37
Symlets	sym5,sym18
Coiflets	coif5,coif14
Biorthogonal	bior1.3,bior5.5
Reverse Biorthogonal	rbio1.3,rbio5.5

**Table 2:** Statistical features of WPT.

Feature	Formula
Mean	$\mu_{n,i}=\frac{1}{m}\sum_{i=1}^{m}C_{n,i}$
Root mean square	$RMS_{n,i}=\sqrt{\frac{1}{m}\sum_{k=1}^{m}C_{n,i}^{2}}$
Variance	$\sigma_{n,i}^{2}=\frac{1}{m-1}\sum_{i=1}^{m}{\left(C_{n,i}-\mu_{n,i}\right)}^{2}$
Energy-to-Shannon ratio	$\xi \left(n\right)=\frac{E(n)}{S_{\mathrm{entropy}\;}\left(n\right)}$

**Table 3:** Classification performance of coif14 and Haar-based analysis using Decision tree (DT) and support vector machine (SVM) classifiers.

Dataset	MW	Balanced Accuracy		Average F1-Score	
		DT	SVM	DT	SVM
DaLiAc	coif14	93.04	88.27	0.92	0.91
	Haar	87.21	79.05	0.87	0.89
HAR70+	coif14	84.64	82.49	0.84	0.84
	Haar	69.63	67.47	0.73	0.7
HARSense	coif14	74.62	76.53	0.75	0.77
	Haar	71.9	73.97	0.72	0.74
Harth	coif14	75.73	76.12	0.75	0.75
	Haar	71.76	71.73	0.69	0.7
mHealth	coif14	91.92	91.55	0.91	0.92
	Haar	88.52	84.04	0.87	0.84
PAMAP2	coif14	95.75	98.64	0.96	0.97
	Haar	88.42	82.42	0.86	0.83
REALDISP	coif14	78.95	76.89	0.77	0.77
	Haar	71.88	72.15	0.72	0.73
WISDM	coif14	84.88	81.9	0.84	0.82
	Haar	74.54	66.57	0.72	0.68

## Data Availability

The experiments used publicly available datasets, which are WISDM [26], HARSense [27], HARTH [28], HAR70+ [29], MHEALTH [30], PAMAP2 [31], REALDISP [32] and DaLiAc [33].
